# Analysis of the Connecting Effectiveness of the Interphase Zone on the Tensile Properties of Carbon Nanotubes (CNT) Reinforced Nanocomposite

**DOI:** 10.3390/polym12040896

**Published:** 2020-04-13

**Authors:** Yasser Zare, Kyong Yop Rhee

**Affiliations:** Department of Mechanical Engineering, College of Engineering, Kyung Hee University, Yongin 446-701, Korea; y.zare@aut.ac.ir

**Keywords:** carbon nanotubes (CNT), polymer nanocomposites, interphase district, percolation onset, mechanical possessions

## Abstract

The establishment of interphase region around nanoparticles accelerates the percolating of carbon nanotubes (CNT) in polymer nanocomposites reinforced with CNT (PCNT), due to the linking productivity of interphase district before the physical connecting of nanoparticles. Therefore, the interphase is an important character in the networks of CNT in PCNT. Here, a simulation study is presented to investigate the interphase connection in the mechanical possessions of PCNT including tensile modulus and strength. A number of models comprising Takayanagi, Ouali, Pukanszky and Callister are developed by the assumption of an interphase district in the CNT excluded volume. The advanced models depict the optimistic influences of reedy and lengthy CNT besides dense interphase on the stiffness and tensile power of nanocomposites. The Pukanszky calculations depict that the interphase strength plays a more noteworthy role in the nanocomposites strength compared to the CNT length.

## 1. Introduction

The carbon nanotubes (CNT) as ideal nanoparticles can add high stiffness and good electrical conductivity to polymer matrices. Additionally, the nanoscale diameter and large aspect ratio of CNT significantly improve the general properties of polymer CNT nanocomposites (PCNT) [1,2,3,4,5,6,7,8,9,10,11,12,13,14,15,16,17]. The van der Waals attraction between nanotubes leads the aggregation/agglomeration in the nanocomposites, which reduces the filler surface expanse and interrupts the networking level [18,19,20]. Additionally, the interfacial communication/bond/area between the polymer medium and particles should be acceptable for an effective load transfer from the polymer matrix to CNT. So, it is important to facilitate the dispersion of nanoparticles at nanoscale and provide good interfacial properties through some techniques of compatibilizing [21,22]. 

The conductivity of PCNT is found after the filler percolation onset in which the nanofiller forms a network, which causes charge transport [23,24,25]. In addition to percolation threshold for conductivity in nanocomposites, an abrupt improvement of modulus/stiffness was found called a mechanical percolation [26,27,28]. The substantial level of shear modulus in the reinforced cellulose whisker films was attributed to mechanical percolation [29]. Therefore, the network of nanoparticles above a particular volume fraction as percolation onset causes a high increment in the mechanical possessions. The vast interfacial region around the nanoparticles and the numerous nanoparticles in a unit volume change the operative possessions of nanocomposites [30,31]. The interfacial area around the nanoparticles produces an intermediate phase as interphase in nanocomposite samples. The interphase is hardly considered by experimental measurements, owing to the minor extent and multifaceted style. Additionally, most available models on the mechanics cannot suppose the interphase effects [18,32,33]. Therefore, several new and developed models were suggested to study the interphase character in the mechanical behavior [34,35,36]. 

The interphase district around nanoparticles can facilitate the establishment of a networked structure in nanocomposites at a low filler portion [37]. This occurrence declines the percolation onset, due to the connection of interphase area. It was suggested that the interfacial communications between the polymer medium and particles manage the percolation of particles with a high aspect ratio and random orientation [38]. Nevertheless, the interphase percolation and its influences on the mechanical possessions have not been considered in the previous works on the nanocomposites. Even though the earlier articles have suggested the strengthening character of interphase, the interphase percolation was not clarified, well. The interphase can quicken the filler percolation making a novel tactic in the interphase and filler network in nanocomposites. In this article, the novel interphase percolation surrounding nanoparticles is defined to progress the available models for nanocomposites mechanical properties. This paper examines the effects of CNT size and interphase possessions on the tensile modulus and strength by Takayanagi, Ouali, Pukanszky and Callister models assuming interphase percolation. The outputs of this paper can conduct the investigators to understand the role of interphase in the percolating of nanoparticles. Moreover, the present article indicates the strengthening and percolating effects of interphase in nanocomposites. 

## 2. Equations and Developed Models

The percolation onset in PCNT supposing the filler and interphase percolation was obtained [39] as:

(1) $$\phi_{p}=\frac{\pi R^{2}l+(4/3)\pi R^{3}}{\frac{32}{3}\pi {\left(R+t\right)}^{3}[1+\frac{3}{4}(\frac{l}{R+t})+\frac{3}{32}{\left(\frac{l}{R+t}\right)}^{2}]}$$

where “*R*” and “*l*” show the CNT radius and length, correspondingly. In addition, “*t*” denotes the interphase depth surrounding CNT. 

Some authors [40] advanced the Takayanagi equation assuming the networking and dispersal of CNT exceeding percolation onset as:

(2) $$E=\frac{\phi_{N}(1-\phi_{f})E_{f}^{}E_{N}^{}+\phi_{N}(\phi_{f}-\phi_{N})E_{m}^{}E_{N}^{}+{\left(1-\phi_{N}\right)}^{2}E_{f}^{}E_{m}^{}}{(1-\phi_{f})E_{f}^{}+(\phi_{f}-\phi_{N})E_{m}^{}}$$

where “$\phi_{f}$” and “$\phi_{N}$” show the volume portions of CNT and nets, correspondingly. Additionally, “*E*_f_”, “*E*_N_” and “*E*_m_” denote the tensile moduli of detached nanofiller, net and polymer medium, correspondingly. The nonappearance of net ($\phi_{N}$ = 0) condenses the advanced model to:

(3) $$E=\frac{E_{f}^{}E_{m}^{}}{(1-\phi_{f})E_{f}^{}+\phi_{f}E_{m}^{}}$$

The percentage of percolated CNT can be roughly expressed [41] by:

(4) $$f=1-\exp[-A\left(\frac{\phi_{f}}{\phi_{p}}-1\right){}^{0.474}]$$

where “A” is constant depending to the net level. The volume portion of filler net is also estimated as:

(5) $$\phi_{N}=\frac{f\phi_{f}}{1-(1-f)\phi_{f}}≅f\phi_{f}$$

By substituting Equations (4) and (5) into Equation (2), the tensile modulus of nanocomposites predicted by Takayanagi model is correlated to the percolation threshold.

Ouali et al. [42] also added the percolation onset to the reverse rule of mixtures as:

(6) $$E=\frac{(1-2\psi +\psi \phi_{f}^{})E_{m}^{}E_{f}^{}+(1-\phi_{f}^{})\psi E_{f}^{2}}{(1-\phi_{f}^{})E_{f}^{}+(\phi_{f}^{}-\psi )E_{m}^{}}$$

where “ψ” parameter depends on “$\phi_{p}^{}$” as:

(7) $$\psi =\phi_{f}^{}{\left(\frac{\phi_{f}^{}-\phi_{p}^{}}{1-\phi_{p}^{}}\right)}^{b}$$

The “b” factor is a percolation exponent, which equals to 0.4 in 3D scheme [42]. Moreover, the relative modulus is defined as E/E_m_.

There are two known models for tensile strength/power of nanocomposites including Pukanszky and Callister. They do not include the “$\phi_{p}$” parameter, but the aspect ratio of nanofiller (α) in these models can be related to “$\phi_{p}$” above the percolation threshold. As a result, they are expressed by “$\phi_{p}$” and the new form of “$\phi_{p}$” (Equation (1)) develops them to show the interphase percolation. 

Pukanszky [43] recommended a model for strength of composites as:

(8) $$\sigma_{R}=\frac{1-\phi_{f}}{1+2.5\phi_{f}}\exp(B\phi_{f})$$

where “σ_R_” is relative strength as σ_c_/σ_m_; “σ_c_” and “σ_m_” show the tensile strength of composite and polymer medium, correspondingly. In addition, “B” as an interfacial factor displays the extent of stress flowing between polymer medium and filler. This model has been acceptably applied for strength of polymer nanocomposites [44,45].

The “*B*” parameter depends on the thickness and strength of interphase by:

(9) $$B=(1+A_{c}d_{f}t)\ln(\frac{\sigma_{i}}{\sigma_{m}})$$

where “*A*_c_” and “*d*_f_” show the specific surface area and density of nanofiller, in that order. Additionally, “σ_i_” is interphase strength. “*A*_c_” for cylindrical rods such as CNT is formulated by:

(10) $$A_{c}=\frac{A}{m}=\frac{A}{d_{f}V}≅\frac{2\pi Rl}{d_{f}\pi R^{2}l}=\frac{2}{d_{f}R}=\frac{4\alpha }{d_{f}l}$$

where “*A*” and “*m*” show the filler surface area and mass, correspondingly. In addition, α = l/*d*, where “*d*” is the diameter of particles. 

Chatterjee [41] suggested a simple equation between “$\phi_{p}$” and “α” as:

(11) $$\phi_{p}^{}\approx \frac{1}{\alpha }$$

By replacing of “α” from Equation (11) into Equation (10), “*A*_c_” can be given by:

(12) $$A_{c}=\frac{4}{d_{f}l\phi_{p}}$$

By substituting of “*A*_c_” from Equation (12) into Equation (9), “*B*” is stated by percolation onset as:

(13) $$B=(1+\frac{4}{l\phi_{p}}t)\ln(\frac{\sigma_{i}}{\sigma_{m}})$$

When “*B*” from above equation is considered in Equation (8), the relative strength is estimated by:

(14) $$\sigma_{R}=\frac{1-\phi_{f}}{1+2.5\phi_{f}}\exp\left((1+\frac{4}{l\phi_{p}}t)\ln(\frac{\sigma_{i}}{\sigma_{m}})\phi_{f}\right)$$

Callister [46] also projected the yield strength of polymer composites by interfacial properties as:

(15) $$\sigma_{R}=1+(\frac{\alpha s}{\sigma_{m}}-1)\phi_{f}$$

where “*s*” denotes the interfacial stress transferring. Even if this model was originally proposed for short fiber composites, it can be effectively used for polymer nanocomposites [47,48]. 

The Callister model can be expanded by percolation onset (Equation (11)) as:

(16) $$\sigma_{R}=1+(\frac{s}{\phi_{p}\sigma_{m}}-1)\phi_{f}$$

which simply connects the nanocomposites strength to percolation onset. When the “$\phi_{p}$” from Equation (1) is replaced into Equation (16), the relative strength above the percolation onset is represented as:

(17) $$\sigma_{R}=1+\left(\frac{s\frac{32}{3}\pi {\left(R+t\right)}^{3}[1+\frac{3}{4}(\frac{l}{R+t})+\frac{3}{32}{\left(\frac{l}{R+t}\right)}^{2}]}{[\pi R^{2}l+(4/3)\pi R^{3}]\sigma_{m}}-1\right)\phi_{f}$$

which explicitly correlates the strength of nanocomposites to matrix, nanoparticles and interphase properties accounting for the interphase percolation.

## 3. Results and Discussion

The expressed models were utilized to investigate the filler and interphase belongings in the mechanical properties of nanocomposites assuming the interphase percolation. In this regard, the mechanical properties were studied as a function of many factors such as the particle size, interphase thickness and strength as well as network modulus. 

**Figure 1:**
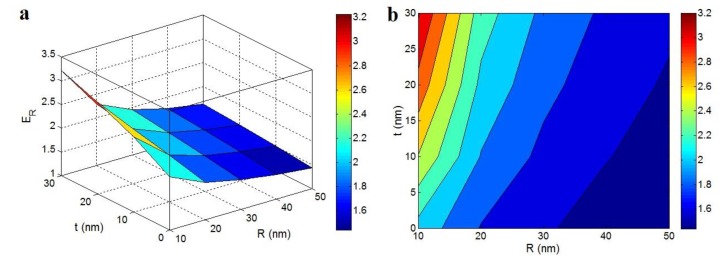
(**a**) 3D and (**b**) contour designs to show the relative modulus at dissimilar “*R*” and “*t*” ranges according to advanced Takayanagi model (*E*_m_ = 2 GPa, $\phi_{f}$ = 0.02, *E*_f_ = 1000 GPa, *l* = 5 μm, *A*= 0.02 and *E*_N_ = 2000 GPa).

Figure 1 revealed the “*R*” and “*t*” roles in the tensile modulus of nanocomposite based on the advanced Takayanagi model and interphase percolation at *E*_m_ = 2 GPa, $\phi_{f}$ = 0.02, *E*_f_ = 1000 GPa, *l* = 5 μm, *A*= 0.02 and *E*_N_ = 2000 GPa. The high ranges of modulus were acquired by reedy nanoparticles and dense interphase. As observed, *E*_R_ = 3.2 was reported at *R* = 10 nm and *t* = 30 nm. However, the high levels of “*R*” and small values of “*t*” negligibly progressed the modulus. Therefore, the modulus of nanocomposites inversely related to the radius of nanoparticles, while the interphase thickness caused a positive effect on the modulus. 

The thin nanotubes could produce a large level of surface part, which grew the interfacial area. As known, the big interfacial area at the polymer–nanoparticles interface promoted the interfacial interaction and improved the modulus. Additionally, the small nanoparticles caused the robust interfacial communications with the polymer medium, owing to the analogous scopes of polymer chains and nanofillers [49]. So, the thin nanotubes caused the sturdy connections amid polymer medium and nanoparticles, which increased the modulus. On the other hand, a denser interphase yielded a superior modulus demonstrating the positive effect of interphase percolation on the modulus. A thick interphase facilitated the connection between interfacial region and formation of the filler network without the physical links between nanoparticles. Therefore, a thicker interphase caused a better network, which caused a better modulus. Nevertheless, thick nanoparticles made a low aspect ratio and small interfacial area. In addition, a thin interphase delayed the formation of a network in the sample, which insignificantly increased the modulus. Accordingly, the thick nanotubes and thin interphase could not enhance the modulus of nanocomposites.

**Figure 2:**
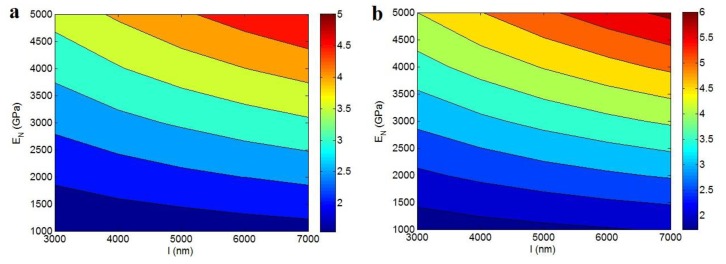
Contour plots for “*l*” and “*E*_N_” roles in the relative modulus by advanced Takayanagi model at (**a**) *t* = 5 nm and (**b**) *t* = 15 nm (*E*_m_ = 2 GPa, $\phi_{f}$ = 0.02, *R* = 10 nm, *E*_f_ = 1000 GPa, *A*= 0.02).

Figure 2 also shows the “*l*” and “*E*_N_” effects on the relative modulus by an advanced Takayanagi model at *E*_m_ = 2 GPa, $\phi_{f}$ = 0.02, *R* = 10 nm, *E*_f_ = 1000 GPa, A = 0.02 and different thicknesses of interphase. The modulus shows a low level at the poor values of “*E*_N_”. However, the uppermost modulus was calculated by the top levels of “*l*” and “*E*_N_”. About 50% improvement in modulus was observed at *E*_N_ < 1200 GPa at *t* = 5 nm (Figure 2a), but nearly 400% growth in modulus was observed at *l* = 7000 nm and *E*_N_ = 5000 GPa at the same interphase. These observations show the desirable effects of filler length and network modulus in the stiffness of nanocomposites. Clearly, lengthy CNT with a high aspect relation and large interfacial area yielded a robust interfacial interaction with polymer media. These factors facilitated the stress moving, which grew the nanocomposites modulus. Additionally, a strong network of nanoparticles could stand the big stress, which caused a high stiffness in the nanocomposite. The polymer matrix with low stiffness could not tolerate the stress loading, but the network of nanoparticles with extraordinary modulus could significantly reinforce the polymer matrix against the high stress. 

Figure 2b displays the “*l*” and “*E*_N_” effects on the relative modulus at a thicker interphase as *t* = 15 nm. The similar roles of these factors in the modulus were also obserevd in this illustration, but the level of modulus increased when the interphase thickness rose. At the same levels of *l* = 7000 nm and *E*_N_ = 5000 GPa, the highest “*E*_R_” at *t* = 5 nm was obtained as 5, while the “*E*_R_” increased to 6 at *t* = 15 nm. As a result, the formation of a denser interphase caused a greater modulus, due to the better properties of network produced by interphase percolation.

**Figure 3:**
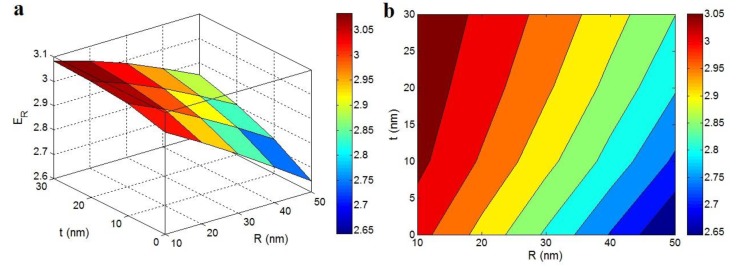
The relative modulus as a function of “*R*” and “*t*” terms by the Ouali model: (**a**) 3D and (**b**) contour plots.

Figure 3 demonstrates the “*R*” and “*t*” influences on the relative modulus by the Ouali model at *E*_m_ = 2 GPa, $\phi_{f}$ = 0.02, *E*_f_ = 1000 GPa and *l* = 5 μm. The relative modulus of about 3 was predicted at low “*R*” and high “*t*”, while the smallest modulus (*E*_R_ = 2.65) was detected at high “*R*” and minor “*t*”. Hence, the relative modulus was inversely and straightly linked with the radius of nanoparticle and thickness of interphase, respectively. These predictions were similar to those of developed Takayanagi model, which confirmed the parameters’ roles in the total stiffness. 

The thin nanotubes show the acceptable dispersion of nanoparticles at the nanoscale in the polymer matrix, because some unwanted occurrences such as an accumulation increase of the radius of particles [50]. On the other hand, a dense interphase can hasten the development of filler net in the nanocomposites, which largely progresses the modulus. Consequently, the Ouali model suitably shows the “*R*” and “*t*” roles in the relative modulus accounting the percolating of interphase. In addition to the percolating effect of interphase, the good reinforcing efficiency of interphase has been well reported [51,52], due to its higher modulus compared to polymer matrix.

**Figure 4:**
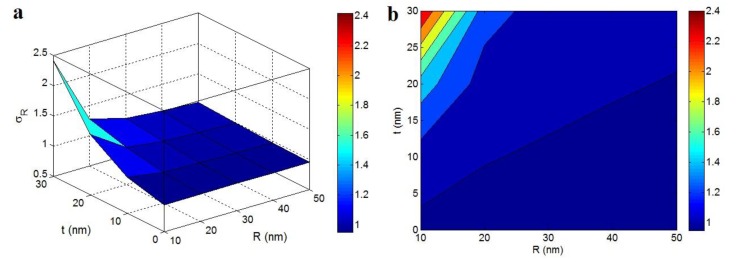
The influences of “*R*” and “*t*” terms on the relative strength assuming the percolating interphase based on the Pukanszky model at (**a**) 3D and (**b**) contour illustrations (σ_m_ = 40 MPa, $\phi_{f}$ = 0.02, σ_i_ = 100 MPa and *l* = 5 μm).

Figure 4 illustrates the “*R*” and “*t*” influences on the relative strength according to Pukanszky model at σ_m_ = 40 MPa, $\phi_{f}$ = 0.02, σ_i_ = 100 MPa and *l* = 5 μm. This model predicted σ_R_ < 1 (the poorer strength of the nanocomposite than the polymer matrix) at high “*R*” and small values of “*t*”. This occurrence demonstrates that the strength of nanocomposites did not improve by big nanoparticles and a thin interphase. However, the improvement of strength started by decreasing in “*R*” and increasing in “*t*”. The highest strength based on the Pukanszky model as σ_R_ = 2.4 was observed by the smallest nanoparticles and the thickest interphase. Hence, the extents of both particles and interphase significantly changed the nanocomposites strength.

Thin nanoparticles provided a high strengthening role in the nanocomposites, because they made a huge specific surface extent (Equation (10)), which significantly increased the interfacial communications. It was indicated that the high surface area of nanoparticles and strong interfacial interaction/adhesion are essential to realize an extraordinary strength in the nanocomposites [44,53,54]. On the other hand, a dense interphase caused a high excluded volume and low percolation onset in the nanocomposites, which accelerated the formation of net by large interfacial region. As a result, the strength of nanocomposites improved by the assumption of interphase percolation in the nanocomposites. Beside the percolation of interphase, a thick interphase (great *t*) significantly improved the strength of nanocomposites by its strengthening nature as shown in the Pukanszky model (Equation (14)).

**Figure 5:**
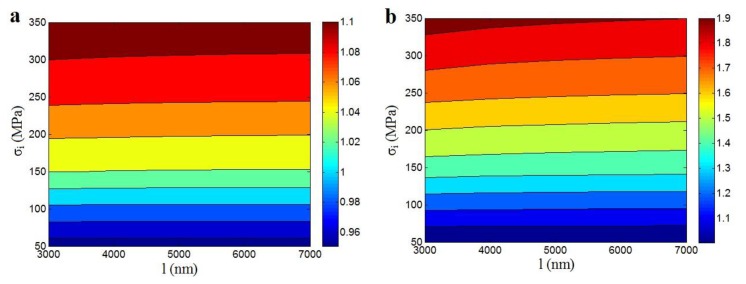
The predictions of Pukanszky model at different levels of “*l*” and “σ_i_” factors at σ_m_ = 40 MPa, $\phi_{f}$ = 0.02, *R* = 10 nm and (**a**) *t* = 5 nm and (**b**) *t* = 15 nm.

Figure 5 also displays the Pukanszky predictions at changed “*l*” and “σ_i_” levels (σ_m_ = 40 MPa, $\phi_{f}$ = 0.02, *R* = 10 nm and *t* = 5 and 15 nm). As observed, the “σ_i_” parameter played a dominant character in the nanocomposites strength. The level of interphase strength directly controlled the strength for nanocomposites, while the CNT length did not affect the nanocomposites power in Figure 5a (*t* = 5 nm). Consequently, the role of interphase strength in the nanocomposites strength was more important than that of the nanotubes length. It demonstrated that the properties of interphase played a chief character in the strength. However, it should be noted that the poor levels of “σ_i_” smaller than 125 MPa could not progress the relative strength. Accordingly, the deprived strength of samples might be related to the low strength of interphase. 

Figure 5b also displays the important character of interphase power in the relative strength, but the nanotubes length can slightly affect the strength at the high ranks of “σ_i_”. Furthermore, the highest level of relative strength was obtained as 1.9 at σ_i_ = 350 and *t* = 15 nm (Figure 5b), while the highest relative strength of 1.1 was obtained at the same interphase strength and *t* = 5 nm (Figure 5a). As a result, the thickness of interphase had a positive effect on the tensile power. As mentioned, the interphase could decrease the percolation volume fraction of nanoparticles in the specimens by contacting of interphase area around the nanoparticles without the direct joining of nanoparticles. Additionally, the interphase induced a strengthening role in the nanocomposites, as reflected in the literature [27,55]. Therefore, the interphase undoubtedly promoted the strength of nanocomposites through strengthening effect and altering the percolation point.

**Figure 6:**
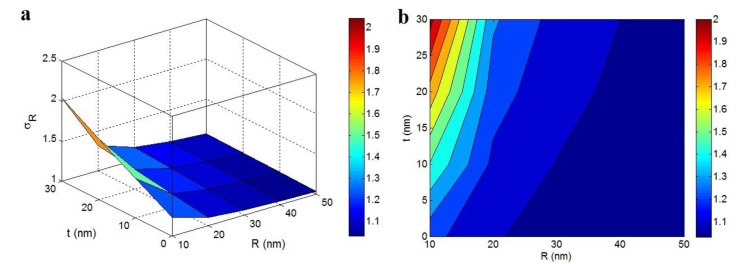
(**a**) 3D and (**b**) contour schemes for the influences of “*R*” and “*t*” on the relative strength expending the Callister model (σ_m_ = 40 MPa, $\phi_{f}$ = 0.02, *s* = 1 MPa and *l* = 5 μm).

The influences of nanoparticles radius and interphase thickness on the relative strength are shown in Figure 6 utilizing the Callister model and interphase percolation. The Callister model calculates the worst strength of nanocomposites at high “*R*” levels. As indicated, the high levels of “*R*” decline the reinforcing effect of nanoparticles through decreasing the specific surface area and weakening the interfacial interaction. As detected in Figure 6, the uppermost strength was seen at the least “*R*” and the highest “*t*”. A great level of “*t*” displays the robust interfacial interaction/adhesion [18,43]. Moreover, a thick interphase easily produced a large net at low concentration of nanofiller. Consequently, the interphase is a vital factor in the possessions of nanocomposites.

**Figure 7:**
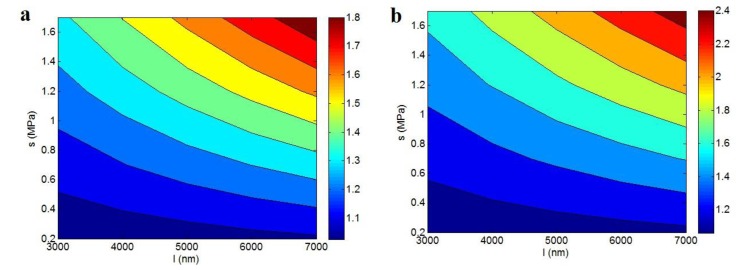
The roles of “*l*” and “*s*” parameters in the relative strength established by the Callister model: (**a**) *t* = 5 nm and (**b**) *t* = 15 nm.

Figure 7 also illustrates the “l” and “s” roles in the relative strength based on Equation (17) at σ_m_ = 40 MPa, $\phi_{f}$ = 0.02, *R* = 10 nm and *t* = 5 and 15 nm. The highest relative strength is acquired by the uppermost levels of “*l*” and “*s*” factors. Therefore, these parameters positively control the relative strength. It is expected, since the nanotubes length as a material parameter determines the levels of aspect ratio and interfacial area. In addition, a high “*l*” parameter increased the level of excluded volume, which decreased the percolation threshold of nanoparticles in the nanocomposites (see Equations (6) and (7)). Furthermore, the “*s*” parameter shows the stress transfer as a function of interfacial interaction/adhesion at the interface. Clearly, a high “*s*” creates an excessive strength in the nanocomposites.

The “*l*” and “*s*” parameters analogously changed the strength of nanocomposites in Figure 7a,b, but the level of improved strength was different depending on the interphase thickness. The relative strength improved from 1.03 to 1.8 in Figure 7a at *t* = 5 nm, while it grew from 1.06 to 2.4 in Figure 7b at *t* = 15 nm. Therefore, a more interphase thickness produced better strength in the nanocomposites. According to the mentioned remarks, the interphase advanced the strengthening effect and percolating of nanofillers, which increased the strength of nanocomposites.

**Figure 8:**
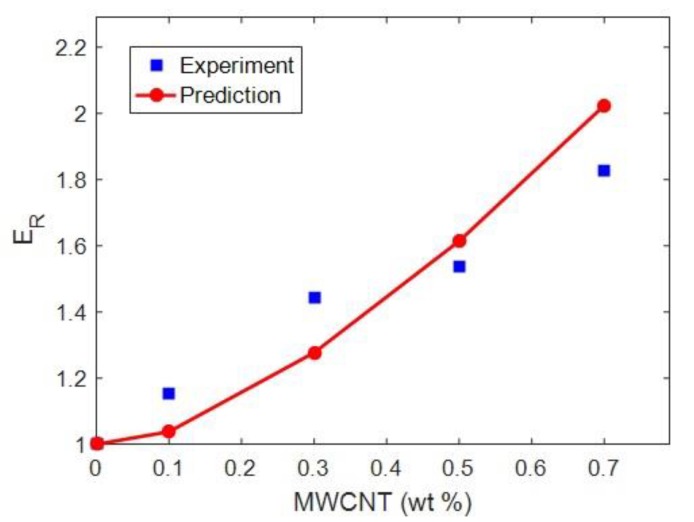
Experimental and theoretical relative modulus by the advanced Takayanagi model (Equation (2) for epoxy/MWCNT nanocomposites [56].

Figure 8 shows the experimental and theoretical relative modulus by an advanced Takayanagi model (Equation (2)) for epoxy/multi wall CNT (MWCNT) nanocomposites [56]. *E*_m_ = 0.52 GPa as well as average *R* = 25 nm and *l* = 50 μm were reported in that paper. In addition, *E*_f_ = 1000 GPa and *A* = 0.02 were considered for calculations. As observed, the experimental and theoretical data show good agreement at all CNT concentrations. This agreement confirms the predictability of the advanced Takayanagi model assuming a percolation threshold. In fact, this model considered the roles of interphase thickness, network modulus and percolation onset in the modulus of nanocomposites. Accordingly, *t* = 5 nm and *E*_N_ = 2100 GPa were obtained producing the percolation threshold of 0.0004 for this sample. These results indicate that this epoxy/MWCNT sample included the interphase region and CNT network based on the suggested equations.

## 4. Conclusions 

The interphase district was assumed in the percolation onset of the nanofiller to develop the Takayanagi, Ouali, Pukanszky and Callister models for tensile properties. These models investigated the effects of some factors attributed to nanoparticles and interphase on the tensile modulus and strength. Both Takayanagi and Ouali models predicted the high ranges of relative modulus by reedy nanoparticles and dense interphase, because reedy CNT cause a big interfacial area and robust interfacial communications with polymer medium. Moreover, a thick interphase facilitates the formation of filler net at lower nanofiller concentration without the filler physical links. The Takayanagi model also revealed the optimistic effects of nanotubes length and net modulus on the tensile modulus. Pukanszky and Callister models predicted the poor strength for nanocomposites at high levels of nanoparticles radius and small values of interphase thickness. However, the highest strength was observed by the smallest nanoparticles and the thickest interphase. The interphase strength played a more significant role in the nanocomposites strength in comparison to CNT length supposing the Pukanszky calculations. Conclusively, the tensile belongings of nanocomposites generally increase through the reinforcing effect of interphase and decreasing the percolation point by interphase area.
